# Challenges in pain assessment and management among individuals with intellectual and developmental disabilities

**DOI:** 10.1097/PR9.0000000000000822

**Published:** 2020-06-16

**Authors:** Chantel C. Barney, Randi D. Andersen, Ruth Defrin, Lara M. Genik, Brian E. McGuire, Frank J. Symons

**Affiliations:** a Gillette Children's Specialty Healthcare, Saint Paul, MN, USA; b Department of Educational Psychology, University of Minnesota, Minneapolis, MN, USA; c Department of Research, Telemark Hospital Trust, Skien, Norway; d Department of Physical Therapy, School of Health Professions, Sackler Faculty of Medicine & Sagol School of Neuroscience, Tel-Aviv University, Israel; e Department of Psychology, University of Guelph, Guelph, ON, Canada; f School of Psychology and Centre for Pain Research, National University of Ireland, Galway, Ireland

**Keywords:** Pain, Discomfort, Intellectual and developmental disabilities, Quantitative sensory testing, Cerebral palsy, Cognitive impairment

## Abstract

**Introduction:**

Intellectual and developmental disabilities (IDD) include conditions associated with physical, learning, language, behavioural, and/or intellectual impairment. Pain is a common and debilitating secondary condition compromising functional abilities and quality of life.

**Objectives:**

This article addresses scientific and clinical challenges in pain assessment and management in individuals with severe IDD.

**Methods:**

This Clinical Update aligns with the 2019 IASP Global Year Against Pain in the Vulnerable and selectively reviews recurring issues as well as the best available evidence and practice.

**Results:**

The past decade of pain research has involved the development of standardized assessment tools appropriate for individuals with severe IDD; however, there is little empirical evidence that pain is being better assessed or managed clinically. There is limited evidence available to inform effective pain management practices; therefore, treatment approaches are largely empiric and highly variable. This is problematic because individuals with IDD are at risk of developing drug-related side effects, and treatment approaches effective for other populations may exacerbate pain in IDD populations. Scientifically, we are especially challenged by biases in self-reported and proxy-reported pain scores, identifying valid outcome measures for treatment trials, being able to adequately power studies due to small sample sizes, and our inability to easily explore the underlying pain mechanisms due to compromised ability to self-report.

**Conclusion:**

Despite the critical challenges, new developments in research and knowledge translation activities in pain and IDD continue to emerge, and there are ongoing international collaborations.

## Abstract 2

Pain is common for individuals with intellectual and developmental disabilities, and we need to accelerate the use of evidence-based approaches to assess and manage pain.

**Key Points**

There is now abundant evidence that individuals with intellectual and developmental disabilities (IDD) experience acute and chronic pain with at least the same frequency as the rest of the population.
Pain assessment tools are available to be used routinely to detect and monitor pain in individuals with IDD.
Important initiatives such as the Global Year for Pain in the Most Vulnerable demonstrate advances in the field, raise awareness, and are likely to bolster efforts in this underserved population.

## 1. Introduction

Developmental disabilities incorporate a diverse group of conditions associated with impairment in physical, learning, language, or behavioral functioning or a combination of these. These conditions begin during the developmental period, may impact day-to-day functioning, and usually last throughout a person's lifetime.^55^ Within that broad grouping, the diagnosis of intellectual disability (previously referred to as “mental retardation” in United States and “learning disability” in United Kingdom) is characterized by significant limitations both in intellectual functioning and in adaptive behavior, which covers many everyday social and practical skills, and originates before the age of 18.^1^ Our focus is to describe the challenges of pain assessment and management in individuals with IDD-related severe communicative, motor, and cognitive impairment.

Research on pain in individuals with IDD is relatively scarce, although pain is often a part of daily life.^46^ A contributing factor for the paucity is likely the routine exclusion of individuals with IDD from pain research, possibly because the numerous functional limitations as well as the underlying neurologic condition frequently confuse the presentation of pain and make it difficult to measure. At the time of writing, there were only 33 scientific articles published specific to pain and individuals with IDD in PubMed (using “intellectual,” “developmental,” “disability” as search terms) in the 5-year period July 2014 to July 2019. In comparison, the terms “pain” and “human” yield over 134,000 publications for the same period. Considering approximately 2% to 3% of the population lives with intellectual disability, autism, or cerebral palsy (CP),^8,45^ the lack of scientific activity-specific pain in the IDD population is striking.

The lack of scientific attention given to pain in individuals with IDD may also be due to long-standing beliefs about pain insensitivity or indifference.^59^ Such beliefs lead to a perspective that individuals with IDD have elevated pain thresholds. The problem with this view—still persisting through to the present—is that studies were rarely designed to assess dimensions considered to be deficient (eg, pain thresholds and reactivity to suprathreshold pain). However, emerging evidence suggests that individuals with IDD may, under certain circumstances, actually be more (not less) sensitive to painful stimuli,^38,44^ have greater pain-evoked potentials,^6,22,54^ and be more likely to experience chronic pain^53^ compared with typically developing peers. Prevalence estimates of chronic pain in IDD average 70% (range = 38%–89%)^60^; these estimates are considerably higher than the general population.^34^ Although the underlying neurologic condition and associated functional limitations may confuse the presentation of pain,^46^ there is little reason to discount or question whether individuals with IDD experience pain, express pain, and are in need of the same pain relieving treatments as their typically developing peers.

In this review, our discussion of the literature is not intended to be exhaustive—readers are directed to informative reviews and practice recommendations provided in the reading list. The purpose of this article is to highlight important points regarding the current state of the evidence specific to pain assessment and treatment among individuals with IDD by focusing on current challenges in the field, active knowledge translation initiatives, as well as key ideas and issues for future attention.

## 2. Challenges in the detection and assessment of pain

Considering the increased exposure of individuals with IDD to injury and the sustained etiology-related pathological conditions that can produce acute and chronic pain,^53,60^ measuring pain among these individuals is essential, yet highly challenging. Although self-report is the most common pain measure used in typically developing individuals, the ability of individuals with IDD to self-report may be limited or absent, depending on the severity of their condition. As the ability of individuals with IDD to use self-report scales is unclear, the use of pain scales with pictures (eg, faces or pyramid scales) or physical items such as blocks to depict pain is preferable.^19,42^ However, even these scales are limited in their valid use among individuals with mild to moderate IDD who have sufficient language and cognitive abilities.^15,21^ Still, self-report should always be considered along with other sources of pain information (eg, behavioral observation, physiological signs, and caregiver report).

The limited communication capabilities of individuals with IDD also limit the use of psychophysical assessment techniques such as quantitative sensory testing (QST) to assess pain sensitivity and pain tolerance.^22^ Such assessment tools require abilities that may be limited or absent among individuals with IDD, such as comprehension of abstract concepts, differentiating between innocuous and noxious stimuli and following instructions. Furthermore, QST is frequently based on reaction time, and individuals with IDD often have slowed reaction time^22^; consequently, their pain threshold may be artificially elevated, erroneously classifying them as hyposensitive to pain. An emerging alternative approach to conventional QST has relied on modifying it by applying standardized and calibrated tactile and noxious stimuli in a stimulus-response application and behavioral measurement approach. The feasibility of the modified QST (mQST) approach has been investigated in children with global developmental delay^4^ and CP^5^ in which differential reactivity to different sensory modalities (eg, light touch, deep pressure, etc.) was documented. Although promising, it does not establish pain threshold but does provide a way of comparing the range of behavioral reactivity in a standardized way across individuals and may have relevance to pain sensitivity as well as sensory function.

Facial and bodily responses to pain and vocalization are perhaps the most intuitive way that caregivers use to identify pain in individuals with IDD.^12^ Frequent pain behaviors noted by parents include moaning, not co-operating, irritable, seeking comfort or closeness, furrowed brow, and difficult to distract or pacify.^11^ Various behavioral scales exist for assessing pain in this population (see Table 1 and reading list). There is no broad consensus as to which scales should be used in routine practice. In some cases, scales have been created without attention to research literature and include items that have not been validated as pain-specific; hence, their evidence base is questionable.^14,57^ Moreover, observing and identifying pain behaviors in individuals with IDD is confounded by many challenges and biases.

Common challenges and biases that should be taken into consideration

(1) Pain is difficult to discern from other conditions or states such as distress, depression, or anxiety, even while observing a familiar person, due to overlap of manifestations.^16^ In typically developing populations, detailed attention to these different states has led to a literature differentiating them.^37^
(2) Diagnostic overshadowing can occur when signs and symptoms of pain are mistakenly attributed to the IDD. Creating personal profiles (eg, hospital passports) to describe the individuals' common signs of pain can assist providers and secondary caregivers in recognizing pain behaviors.
(3) Facial and other behavioral expressions of pain are primarily determined by long established biological dispositions, meaning these expressions are inherently consistent across populations. However, facial and behavioral expressions of pain are also shaped by individual factors such as probable, often unknown, differences in central nervous system structure and function associated with the disability as well as situational factors such as the immediate surroundings, caregiver behavior, and culture.^47^ The influence of unique individual and situational factors on pain expression in individuals with IDD is not well understood.
(4) Individuals with IDD may exhibit idiosyncratic and typical pain behaviors (eg, self-injury, moaning, or facial changes) when they do not have pain—making it difficult for caregivers to discern signs of pain.^12^
(5) As in typically developing samples, observers may not be sufficiently sensitive to the magnitude of pain the individual with IDD experiences. Observers, including parents, are likely to systematically underestimate patients' suffering.^10,50^ Interestingly, underestimation bias was more pronounced in more experienced caregivers than in inexperienced ones.^39^ Underestimation of pain is especially problematic when self-report is not available or reliable.
(6) Observers may be subject to stereotyped beliefs about individuals with IDD in relation to reduced capacity to experience pain,^10,17^ or it is plausible that observers may overestimate pain based on the fact that in many studies on individuals with IDD, observers are not blind to the application of the painful stimulus (eg, venipuncture).

**Table 1:**
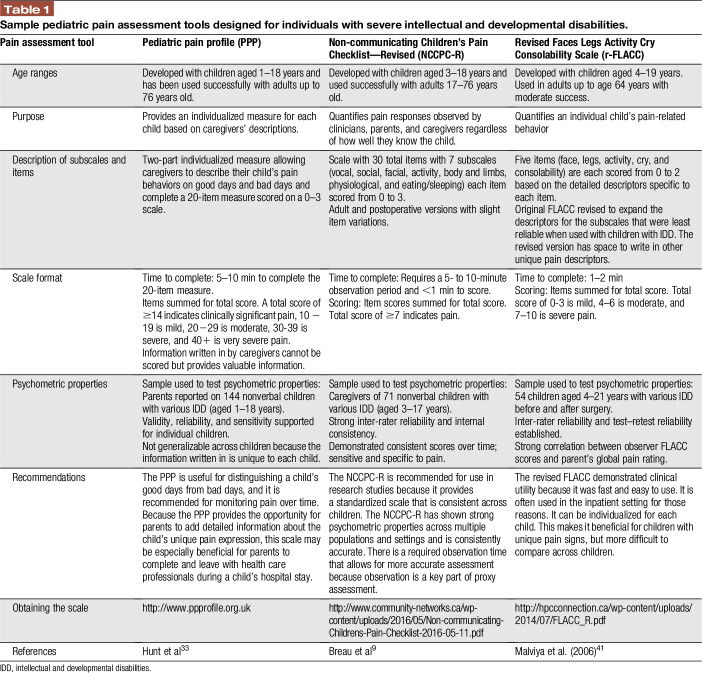
Sample pediatric pain assessment tools designed for individuals with severe intellectual and developmental disabilities.

Mistaken beliefs about pain behavior and pain sensitivity among individuals with IDD resulting from the aforementioned biases may lead to delayed diagnosis and inadequate pain treatment. Studies triangulating methods of assessment to provide convergent validity, such as self-report, psychophysical methods (eg, QST), and caregiver report, may provide the most reliable way of identifying pain in individuals with IDD. Considering that behavior among individuals with IDD may be difficult to interpret, monitoring an individual's “typical behavior” when seemingly pain-free may help to identify changes in behavior associated with pain. Educating caregivers in evidence-based pain assessment tools and in possible cognitive biases may improve pain assessment, although this remains to be determined.

## 3. Challenges in pain management

There is very limited evidence on effective pain management practices for individuals with IDD.^63^ Most studies have been conducted in pediatric populations with poor representation of adolescents and older adults with IDD or of specific disorders. Many of the studies provide low levels of evidence based on study design (eg, case studies and case series) or were assessed to have moderate or weak quality because confounding variables were not controlled, and/or statistical evaluations were not described.^49^ Studies evaluating multidisciplinary interventions, considered the gold standard approach, are virtually nonexistent.^49^ Few treatment protocols or guidelines exist, and management is often highly dependent on the practitioner.^25^ However, where implementation of a standardized approach was feasible, marked improvements in nonverbal children with severe neurological impairments were seen.^58^ Pharmacological and nonpharmacological approaches and additional treatment considerations will be reviewed below.

### 3.1. Pharmacotherapy

When treating individuals with IDD with pharmacotherapy, a number of unique factors should be considered. Overall, individuals with IDD are more at risk of developing drug-related side effects as immature regulation of autonomic reactions, low nutritional status, low liver and kidney functioning, and the concurrent use of multiple drugs may influence both the effectiveness of treatment and the risk of side effects.^3,53^ In addition, condition-specific anatomic and physiologic features could represent additional risk factors (eg, scoliosis can contribute to hypoventilation or airway obstruction).^52^ The presumed difference in pharmacodynamics between individuals with and without IDD highlights the pressing need for pharmacokinetic studies in this population.^61^ A few controlled trials have attempted to determine the best pharmacological approach for pain in children with CP.^52^ However, medication selection continues to be guided by safety and efficacy information from other populations^32^ although such evidence appears to be lacking, even in typically developing children.^23^

### 3.2. Nonpharmacological approaches

Several authoritative reviews have shown that nonpharmacological approaches to pain management are effective at reducing pain burden in the general population.^24,65^ However, little research has examined the effectiveness of such approaches in individuals with IDD. The use of psychotherapy is growing as part of the treatment of conditions such as depression and anxiety in IDD^18,62^ but only a few studies have examined psychological treatments for pain management. For example, case studies using modified cognitive behavioral therapy showed preliminary evidence of benefit in a number of domains,^40,43^ but behavioral components of the intervention were more easily understood than cognitive components.^43^ Subsequently, a protocol was developed for the first clinical trial of cognitive behavioral therapy to manage menstrual pain in women with IDD^36^ based on a manualized treatment program. These studies suggest a role for modified psychological therapies, but more evaluation and refinement of the therapeutic content are needed.

### 3.3. Prevention strategies

Prevention is the most powerful pain-reducing approach. Prevention strategies are undoubtedly underutilized in IDD, for example, preventive measures were seldom taken to reduce pain incurred during daily care activities.^7^ Similarly, hip dislocation is common in CP and is known to be associated with chronic nociceptive pain.^32^ However, the rate of dislocation can be reduced to almost zero if children are included from an early age in a surveillance program (ie, repeated radiographic and clinical examinations) with preventive treatment for hips that are displacing.^30^

### 3.4. Register data

Registers may also inform the prevention of other types of acute and chronic pain. Registers are large databases created for the purpose of collecting uniform observational data that can be used to inform specific clinical and research agendas.^28^ For example, register data showed that early treatment of spasticity in CP (using continuous intrathecal baclofen infusion and botulinum toxin treatment) and early nonoperative treatment of contractures reduced the need for orthopedic surgery for contracture or torsion deformity, and the need for multilevel procedures seemed to be eliminated.^31^ This is important, as procedures and surgeries are sources of acute pain in children with CP and may contribute to increased risk for chronic postsurgical pain. Unfortunately, apart from the Scandinavian countries (Norway, Sweden, and Denmark) and a few others (Scotland, Australia, and Iceland), these types of registers with systematic follow-up of children and adolescents with CP are rare.

### 3.5. Caution in recommendations

Although it may be tempting to consider adopting treatments from the general population, findings from other populations cannot necessarily be safely and effectively adopted in the IDD population or even findings from one IDD diagnosis/subgroup to another. For example, a review of the literature found limited, yet positive support for the effectiveness of exercise on pain in ambulatory adults with CP,^63^ but a subsequent study found the same exercise program exacerbated the pain of nonambulatory adults with CP.^64^ This highlights the need to systematically test available interventions in different IDD populations and subgroups.

## 4. Challenges in research

There are many unanswered questions remaining in relation to optimizing assessment and management of pain in individuals with IDD and much more research is required. However, conducting research in this area is not without its challenges, some of which are identified here.

### 4.1. Proxy-report biases

For individuals with the most severe and profound IDD, self-report of pain is not feasible. In the absence of self-report, parent or other caregiver proxy report is relied on. We have very little scientific understanding of the intrapersonal and interpersonal factors as well as the social/cultural factors that may influence the caregiver's ability to provide accurate pain ratings. Obtaining a proxy report of pain is sometimes the only feasible option, and this approach is certainly superior to not assessing pain. The accurate assessment of another individual's internal physiological and psychological state is not without serious challenge and has been compared to a “mind reading” task.^29^ Obviously, assessing another's pain experience requires skill, sensitivity, and astute judgment; even then, it can be difficult to distinguish pain from other expressions such as distress or anxiety. Caregiver assessment scores may be aligned more closely with their own psychosocial distress (eg, depression and catastrophizing) than with the experience of the person for whom they are reporting.^20^ Future pain research in IDD should include evaluations of the psychosocial characteristics of the proxy to further understand the importance of these factors. In addition, proxy report might be compared with direct observation of nonverbal behaviors to better establish the properties of proxy report and to determine the sources of observer judgments. Development of creative methodological approaches to more objectively assess pain experience and somatosensory function in individuals with IDD (eg, the mQST approach described above) would provide additional avenues to understand pain in this especially vulnerable population where self-report is not an option. The reliability and validity of self-report questionnaires for those with mild to moderate IDD also needs to be established.

### 4.2. Identifying valid outcome measures for use in treatment trials

Treatment trial outcomes are not only dependent on the participants and the intervention under study, but on the selected outcome measures and their measurement properties. The quality of the evidence supporting observational pain scales differs.^2^ The use of measures with questionable or inadequate measurement properties may result in overestimation or underestimation of treatment effects. To take one example, the outcome measure needs to be validated in the sample and setting under study. In one study,^56^ parent-reported Non-Communicating Children's Pain Checklist—postoperative version^9^ scores were used to evaluate procedural pain. Although the measure is validated for the population under study, it has not been validated for procedural pain. There is a need to identify a core set of outcome measures for pain in individuals with IDD, recognizing that the way in which outcomes are operationalized may differ for those who are verbal compared with those who are nonverbal.

### 4.3. Adequately powered studies

Given the wide variety of causes of intellectual disability, comorbidities, and comedication in individuals with IDD, it is not easy to conduct intervention studies with sufficient sample sizes. Solutions may include using collaborative networks across institutions and countries to be able to recruit large samples or to set up national or multinational registers to systematically collect data on pain in these populations. Although there are many longitudinal data registers, they may contain limited and/or variable data on pain, and they may not necessarily include individuals with IDD. Having a core set of outcome measures and an internationally agreed upon research agenda would help address these challenges.

### 4.4. Unexplored pain mechanisms

It is fair to say that almost all pain research in IDD to date has focused on pain prevalence, burden, and the development of pain measurement scales, with minimal work on understanding pain mechanisms. Although the importance of documenting the prevalence and burden of pain in IDD cannot be overstated, the lack of attention to mechanisms of pain makes it unlikely that we will get any closer to personalizing pain treatment for individuals living with chronic pain and IDD. Current approaches to pain assessment in individuals with IDD, essentially measure pain presence (vs absence) and indicate pain intensity/severity. This would usually be sufficient for clinical populations living with intact motor, communicative, and cognitive function (ie, those who can self-report information about changes in intensity, location, temporal features, perceptual qualities, and body locations). However, it presents a serious clinical challenge for individuals with IDD with complex communication needs and chronic health conditions who cannot easily (or for many, never) self-report their pain. To better understand the physiological aspects of pain in IDD, there is a need for a shift to developing or repurposing existing mechanism assessment methods (eg, mQST described above) to provide reliable and valid information specific to pathophysiological processes contributing to pain.

## 5. Challenges in knowledge translation to applied settings

Research in the field of pain and IDD moves slowly, and moving research into clinical practice takes even longer. One of the inherent researcher-based challenges relates to knowledge translation outcome evaluation. Without valid and reliable measures specific to pain in IDD populations, the impact of interventions or knowledge translation efforts will be difficult to understand. As such, researchers are encouraged to contribute to the development of new measures. For example, a new questionnaire that examines the respondents' knowledge of pain practices in individuals with IDD, the Questionnaire for Understanding Pain in Individuals with Intellectual Disabilities—Caregiver Report Revised, is under development (Genik, Zaretsky, Freedman-Kalchman, and McMurtry, in preparation). This measure was informed by pre-existing evidence and the International Association for the Study of Pain's Core Curriculum.^35^ When developing these measures, it will be important to ascertain the outcomes that are most important to stakeholders, as well as the most accurate and feasible measurement approaches. For example, direct observation may be more appropriate than questionnaires for behavioral outcome measurement; however, in community contexts, this approach may not be feasible. Stakeholder-informed research may identify which research outcomes are most valuable to stakeholders and which approaches may be most appropriate for a given context.

## 6. New developments in knowledge translation to applied settings

### 6.1. Pain assessment tools

Although there has been significant work on developing various pain assessment tools in IDD, there is little evidence of widespread clinical adoption of evidence-based pain assessment. Projects to improve pain practices for individuals with IDD have emerged. In 2014, Holland Bloorview's Chronic Pain Assessment Toolbox for Children with Disabilities (hereafter “toolbox”) was created,^48^ leading to the adoption of standardized pain assessment practices in 10 ambulatory clinics across 2 tertiary hospitals by 2016. The implementation of the toolbox included: (1) informing key stakeholders, (2) provider education sessions, (3) building consensus on which tools to use, (4) obtaining permission to use the tools, (5) trialling chosen tools, (6) developing data storage and auditing processes, and (7) creating patient materials to inform and empower patients/families. Significant changes in pain screening and assessment practices occurred across institutions, with the percentage of patients having a completed pain assessment increasing from <2% pre-toolbox to ≥53% post-toolbox implementation. Item level and total pain scores are used in real time to navigate clinical decisions about each patient's care. In addition, the pain assessment data are available in aggregate to answer important research questions.

### 6.2. Virtual reality

The use of virtual reality (VR) to distract patients during medical procedures for typically developing populations is rapidly growing, and findings suggest VR effectively reduces pain and anxiety with few side effects.^66^ Implementation of VR at Gillette Children's Specialty Healthcare in Minnesota, United States, has been introduced for individuals with IDD; early use has focused on patients who are, at a minimum, able to communicate verbally to have VR removed and/or remove the headset themselves. Virtual reality has been used successfully with individuals with IDD during botulinum toxin injections (with and without nitrous oxide), venipuncture, casting, and for postoperative pain. Controlled research trials to determine the effectiveness of VR in IDD are ongoing.

### 6.3. Pain education for secondary caregivers

Parents have reported the need for the professionals supporting their children to be skilled and knowledgeable when it comes to pain.^13^ Recent work has begun to share existing knowledge with secondary caregivers who support children with IDD in community contexts such as in respite settings or at school. Preliminary pain-related outcomes have demonstrated improved knowledge, altered beliefs, and intention for knowledge application.^26,51^ For example, the empirically informed *Let's Talk About Pain* program—based on the International Association for the Study of Pain's Core Curriculum^35^—educates respite workers supporting children with IDD about what pain is and different ways to assess and manage it.^26^ This program has been successfully piloted and received highly positive endorsements from program participants.^26^ A randomized controlled trial of this program exploring training impact immediately after training completion and at 4- to 6-week follow-up is currently underway. The development of relevant and suitable tools may also help to facilitate communication and knowledge sharing between parents and other caregivers. For example, the empirically informed *Caregiver Pain Information Guide* is designed to be completed by parents to inform secondary caregivers about their child's pain.^27^ This resource probes for information such as a child's common pains, pain expression, and pain management approaches. Initial results from a feasibility/usability study with parents and respite care providers were positive.^27^

## 7. Discussion and implications

Pain is a common condition experienced by individuals with IDD.^46^ Pain often goes under-recognized and poorly managed, in part, due to the scarcity of evidence available to inform care in this vulnerable population. Pain assessment tools for individuals with IDD have been developed^48^ and provide valuable information to direct care, but further reliability and validity evidence is needed. Although there is little empirical evidence to inform pain management in IDD, providers should be aware that (1) pain prevention strategies (eg, hip surveillance programs^30^ and early treatment of spasticity^31^) are vital, (2) individuals with IDD are at greater risk for drug-related side effects,^3^ (3) pain management strategies designed for other populations are not necessarily helpful for individuals with IDD,^64^ and (4) nonpharmacological interventions, with little risk for adverse events, may compliment traditional pain management approaches.^40,43,66^

## 8. Conclusion

Despite the many challenges in the field of pain in IDD, studies to date have demonstrated that individuals with IDD are sensitive (possibly more sensitive) to pain,^22,38,44^ have greater pain evoked potentials,^6^ and seem more likely to experience chronic pain.^34^ Appropriate pain assessment measures specific to IDD have been developed and validated, knowledge translation tools for clinical implementation exist,^26,48^ and initial studies have assessed psychological treatments to manage pain.^62^ To continue moving beyond the challenges, cultivation of greater scientific effort must be encouraged, including support for early-career investigators and clinician scientists in the field and broadening study inclusion criteria to include individuals with IDD whenever practical. Further research is needed to assess the psychometric properties of pain assessment tools in specific IDD populations, to create and test unique methodologies to supplement self- and proxy-report of pain and investigate underlying mechanisms (eg, modified QST, biomarkers, etc.), to launch treatment trials, and to generate multisite studies and national registers to improve sample sizes.

## Disclosures

The authors have no conflicts of interest to declare.
